# Melatonin protects against ovarian damage by inhibiting autophagy in granulosa cells in rats

**DOI:** 10.1016/j.clinsp.2022.100119

**Published:** 2022-10-01

**Authors:** Yan Liu, Xiaohe Zhu, Chunli Wu, Yan Lang, Wenjie Zhao, Yanmin Li

**Affiliations:** a Department of Gynecology, Weifang People's Hospital, Weifang, Shandong, China; b Department of Obstetrics, Weifang People's Hospital, Weifang, Shandong, China; c Department of Reproductive Medicine, Weifang People's Hospital, Weifang, Shandong, China

**Keywords:** Autophagy, Effect, Melatonin, Ovarian Granulosa Cells, Ovarian damage

## Abstract

**Objectives:**

This study sought to further verify the protective mechanism of Melatonin (MT) against ovarian damage through animal model experiments and to lay a theoretical and experimental foundation for exploring new approaches for ovarian damage treatment.

**Method:**

The wet weight and ovarian index of rat ovaries were weighted, and the morphology of ovarian tissues and the number of follicles in the pathological sections of collected ovarian tissues were recorded. And the serum sex hormone levels, the key proteins of the autophagy pathway (PI3K, AKT, mTOR, LC3II, LC3I, and Agt5) in rat ovarian tissues, as well as the viability and mortality of ovarian granulosa cells in each group were measured by ELISA, western blotting, CCK8 kit and LDH kit, respectively.

**Results:**

The results showed that MT increased ovarian weight and improved the ovarian index in ovarian damage rats. Also, MT could improve autophagy-induced ovarian tissue injury, increase the number of primordial follicles, primary follicles, and sinus follicles, and decrease the number of atretic follicles. Furthermore, MT upregulated serum AMH, INH-B, and E2 levels downregulated serum FSH and LH levels in ovarian damage rats and activated the PI3K/AKT/mTOR signaling pathway. Besides, MT inhibited autophagic apoptosis of ovarian granulosa cells and repressed the expression of key proteins in the autophagic pathway and reduced the expression levels of Agt5 and LC3II/I.

**Conclusions:**

MT inhibits granulosa cell autophagy by activating the PI3K/Akt/mTOR signaling pathway, thereby exerting a protective effect against ovarian damage.

## Highlights

• Melatonin (MT) plays a similar role in ovarian damage as 3-MA does.
• MT confers protection against ovarian damage *in vivo* and *in vitro*.
• MT exerts its protective effects by inhibiting the PI3K/AKT/mTOR signaling pathway.

## Introduction

Premature Ovarian Insufficiency (POI), also known as premature ovarian failure, is a follicular dysfunction caused by genetic, immune, and environmental factors that seriously affect the endocrine function and fertility of women.^1^, ^2^, ^3^, ^4^ Currently, the incidence of POI is increasing every year, thereby markedly impacting patient lives. Women of reproductive age who suffer from POI have a high risk of infertility, amenorrhea, and early menopausal syndrome, which seriously affects their quality of life and causes great pain.^5^, ^6^, ^7^ POI is typically characterized by menstrual disturbances, increased gonadotropin levels, and decreased estrogen levels before age 40.^3^^,^^8^, ^9^, ^10^ High gonadotropin levels and low estrogen levels predispose patients to the perimenopausal syndrome, such as vasomotor symptoms, anxiety, and neurological symptoms such as memory loss.^11^, ^12^, ^13^ Moreover, the downregulation of estrogen levels can cause osteoporosis and cardiovascular system disorders in patients with POI.^14^^,^^15^ The treatment options for POI include hormone replacement therapy, egg donation, and ovarian transplantation,^5^^,^^16^ which have been markedly hampered by risks of complications and infections, ethical issues, and limited success in improving ovarian function and fertility. Furthermore, ovarian damage is an important pathogenic factor in POI. Therefore, it is important to elucidate the causes and mechanisms of ovarian damage and develop effective treatment strategies.

Melatonin (MT) is an amine hormone produced by the pineal gland in mammals and humans that is secreted in the ovaries and placenta.^17^^,^^18^ MT exerts its antioxidant effects by scavenging free radicals and reducing oxidative stress injury in human granulosa cells and oocytes.^19^^,^^20^ In mice treated with MT, MT was found to restore oocyte meiosis through its antioxidant and anti-apoptotic effects, as well as promote the maturation of oocytes and prevent the decline of ovarian function.^21^^,^^22^ Previous findings suggest that MT treatment is an effective method for inhibiting premature ovarian failure.^23^ Also, MT may act direct on the ovaries through MT1 and MT2 receptors.^24^^,^^25^ The potential mechanism of MT is to inhibit the production of reactive oxygen species and protect the follicular development process. All studies suggest that treatment with MT is an effective method to inhibit ovarian damage; however, its specific mechanism of action is unclear and requires further studies.

## Materials and methods

### Grouping

All SD rats were randomly divided into the following five groups (*n* = 20 in each group): (1) Control group: normal rats were injected intraperitoneally with an equal volume of saline daily; (2) Model group: rats were injected intraperitoneally with tripterygium glycosides (75 mg/kg/1d) for 14 days to establish the ovarian damage model, followed by daily intraperitoneal injection of an equal volume of saline for 14 days; (3) 3-MA group: ovarian damage model rats were injected intraperitoneally with 3-MA (15 mg/kg/1d) for 14 days; (4) RAPA group: ovarian damage model rats were injected intraperitoneally with RAPA (1.5 mg/kg/1d) for 14 days; and (5) MT group: ovarian damage model rats were injected intraperitoneally with MT (10.0 mg/kg/1d), which was prepared with saline containing 5% ethanol, for 14 days. The animals were fed standard chow and housed in 25°C temperature-controlled rooms with 40–70% relative humidity with the minimum noise level.^26^ All applicable international, national, and/or institutional guidelines for the care and use of animals were followed. This study was approved by Weifang People's Hospital (protocol number: XJTU2AF201-08).

### Ovarian tissue collection

Rats were subjected to fasting at 21:00 on the day of completion of the pharmacological intervention and were anesthetized with sodium pentobarbital solution the following day. After rats were completely anesthetized, the abdominal cavity was opened with sterile surgical scissors, and the organs were gently plucked to one side to enable exposure of the abdominal aorta to the field of view. Blood samples (2 mL) were collected with a disposable needle and left to stand at room temperature for 30 min. Thereafter, blood was centrifuged at 4 °C for 15 min at 3000 rpm. The ovaries were stripped from the surrounding adipose and connective tissues under aseptic conditions, removed and weighed separately. One side of the ovary was immersed in 4% paraformaldehyde solution, and the other side was stored at −80 °C.

### HE staining

The ovarian tissues were sectioned into 5 × 5 × 2 mm tissue blocks, washed with saline, placed in 4% formaldehyde solution for 30‒50 min, and dehydrated with a concentration gradient of ethanol. After the above treatment, the tissue blocks were removed and soaked in a mixture of anhydrous ethanol and xylene for 2h, followed by treatment with pure xylene for 1.5 h. The tissue blocks were placed in ½ paraffin wax + ½ xylene solution and left in an oven at 40 °C for 40 min. The tissue blocks were removed and placed in paraffin wax I (30 min) and paraffin wax II (40 min). Thereafter, the tissue-embedded wax blocks were dried in a constant temperature oven. Histopathological sections of rat ovaries were prepared using a microtome, where the thickness of each section was approximately 5 μm. After the tissue sections were dewaxed, rinsed, hematoxylin stained, eosin stained, and sealed, the sections were photographed and analyzed using the Image Pro Plus 6.0 system.

### Detection of apoptotic cells by flow cytometry

The isolated granulosa cells of each group were digested with trypsin, washed with PBS, and treated with 5 μL Annexin V-EGFP working solution. Thereafter, 5 μL propidium iodide working solution was added and mixed well. The cells were allowed to stand for 5‒10 min at room temperature in the dark and detected by flow cytometry within 1 h. Green fluorescence detection of Annexin V-EGFP was performed via the FITC channel (FL1), and red fluorescence detection of propidium iodide was performed via the PI or FL3 channels.

### Total protein extraction

Rat ovarian tissue was cut with ophthalmic scissors, homogenized on ice for 20 min, and transferred to a centrifuge tube for centrifugation at 15,000 rpm for 15 min at 4 °C. The supernatant was transferred to a new EP tube, and the grouping and volume of supernatant were recorded separately; 5× of buffer was added to the supernatant, and the mixture was heated at 99 °C for 10 min to denature the protein. The resulting sample was stored at −20 °C.

### Immunoblotting

Tissue samples were electrophoresed on SDS-polyacrylamide gels, transferred to 5% skimmed milk, and blocked on a shaker for 1h at room temperature. After blocking, monoclonal antibodies, such as LC3II, LC3I, Agt5, p-PI3K, p-AKT, p-mTOR, and GAPDH were added (1:1000). The samples were incubated overnight with the primary antibodies on a shaker at 4 °C. Thereafter, the corresponding secondary antibodies were added (1:2000) to the samples and incubated for 1h at room temperature. The color was developed by chemiluminescence. The protein grayscale was scanned and analyzed using ImageJ software.

### Data analysis

In this study, data were processed and analyzed using SPSS 19.0. All experiments were repeated at least three times independently. The figures were plotted using GraphPad Prism 6.0 (GraphPad Software Inc., USA) and are indicated as mean ± SD. The student's *t*-test was used for continuous variables. Statistical significance was set at *p* < 0.05.

## Results

### Effect of MT on ovarian wet weight and ovarian index in rats

The left and right ovarian wet weights of rats were highest in the control group (*p* < 0.05) and lowest in the model group (*p* < 0.05). The left and right ovarian wet weights of rats in the mTOR inhibitor (RAPA) group were higher than those in the model group (*p* < 0.05), while the ovarian wet weights of rats in the cellular autophagy inhibitor (3-MA) group and MT group increased compared with those in the RAPA group (*p* < 0.05). There was no significant difference in ovarian wet weight between the 3-MA and MT groups (*p* > 0.05). A similar trend was observed for ovarian indices, with the control group having the highest ovarian index (*p* < 0.05) and model rats possessing the lowest ovarian index (*p* < 0.05). The RAPA group showed a higher ovarian index than the model group (*p* < 0.05), and the 3-MA and MT groups exhibited a higher ovarian index than the RAPA group (*p* < 0.05). The ovaries of the 3-MA and MT groups were not significantly different (*p* > 0.05) between the 3-MA and MT groups (Table 1). These results suggest that the cellular autophagy pathway is associated with ovarian wet weight and ovarian index in rats. Further, MT significantly increased the ovarian wet weight and ovarian index.

**Table 1:** Ovarian wet weight, ovarian index, and follicle numbers of rats in each group.

Group	n	Left ovary wet weight (mg)	Right ovary wet weight (mg)	Ovarian index (mg/g)	Primordial follicle (n)	Primary follicles (n)	Antral follicle (n)	Atretic follicles (n)
Control group	19	51.43 ± 6.92	47.67 ± 5.70	0.35 ± 0.03	9.61 ± 4.30	5.75 ± 2.33	6.51 ± 3.00	2.51 ± 1.50
Model group	19	16.43 ± 5.26^a^	15.78 ± 6.78^a^	0.13 ± 0.03^a^	2.78 ± 1.30^a^	2.33 ± 1.22^a^	1.73 ± 0.83^a^	8.87 ± 3.52^a^
3-MA group	18	30.67 ± 7.47^a^^b^	28.88 ± 4.91^a^^b^	0.23 ± 0.02^a^^b^	6.69 ± 2.12^a^^b^	4.91 ± 2.23^b^	3.97 ± 2.00^a^^b^	5.16 ± 2.64^a^^b^
RAPA group	19	21.86 ± 6.77^a^^b^^c^	19.44 ± 5.90^a^^b^^c^	0.18 ± 0.03^a^^b^^c^	4.22 ± 2.64^a^^b^^c^	3.16 ± 1.56^a^^c^	2.62 ± 1.58^a^^b^^c^	7.25 ± 3.15^a^^b^^c^
MT group	18	29.50 ± 5.24^a^^b^^d^	28.50 ± 4.50^a^^b^^d^	0.22 ± 0.01^a^^b^^d^	6.22 ± 1.91^a^^b^^d^	4.86 ± 2.59^b^^d^	3.14 ± 1.73^a^^b^^d^	4.84 ± 2.36^a^^b^^d^

Ovarian index, Wet weight of bilateral ovaries (mg)/rat body weight (g) × 100%.

a p compared with the control group, *p* < 0.05.

b p compared with the model group, *p* < 0.05.

c p compared with the 3-MA group, *p* < 0.05.

d p compared with the RAPA group, *p* < 0.05.

### Effects of MT on ovarian histomorphology and follicle number in ovaries

**Fig. 1:**
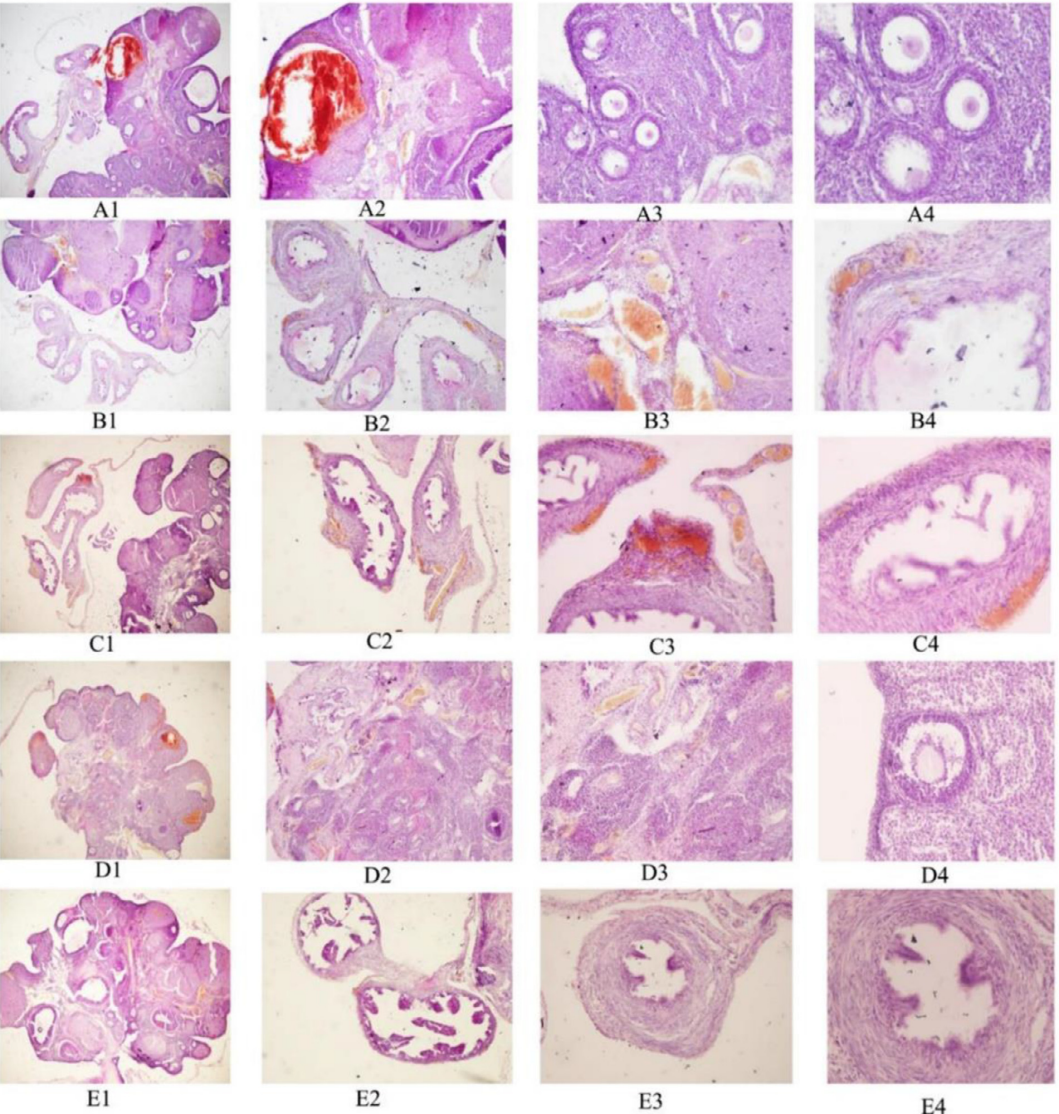
**HE staining of ovarian tissue sections of rats in each group.** A1–A4 represent the MT group; B1–B4 represent the RAPA group; C1–C4 represent the 3-MA groups; D1–D4 represent the model groups; E1–E4 represent the control groups; Nos. 1–4 correspond to magnifications of 4 ×, 10 ×, 20 ×, and 40 ×, respectively.

Ovarian tissue samples were collected from each group of rats and pathological sections were prepared. The morphology of the ovarian tissue in each group was observed by hematoxylin and eosin staining, and the number of follicles in each group was counted. Compared with the control group, the ovarian tissues of the model, 3-MA, RAPA, and MT groups showed different degrees of atrophy, with the model group displaying the highest degree of atrophy. Further, the number of follicles at all levels was significantly reduced, the follicular structure was destroyed, and the corpus luteum was small and poorly developed. Compared with the model group, the atrophy of ovarian tissues in the 3-MA, RAPA, and MT groups was improved to different degrees. Antral follicles, primordial follicles, primary follicles, secondary follicles, and mature follicles were observed in the visual field. Typical structures, such as egg cells, cumulus, zona pellucida, and radial corona could also be found (Fig. 1). The number of granulosa cells wrapped around the periphery of the follicles in these groups was more than that in the model group, showing a regular structure. Moreover, the number of corpora lutea was significantly better developed than that in the model group. The number of primordial follicles, primary follicles, and antral follicles was the highest in the control group and lowest in the model group. The number of primordial follicles, primary follicles, and antral follicles in the RAPA group were higher than those in the model group (*p* < 0.05). Further, the numbers of primordial follicles, primary follicles, and antral follicles in the 3-MA and MT groups were higher than those in the RAPA group (*p* < 0.05). No significant differences were found between the 3-MA and MT groups (*p* > 0.05). Further, the number of atretic follicles was highest in the model group and lowest in the control group. The number of atresia follicles in the RAPA group was less than that in the model group (*p* < 0.05). The number of atretic follicles in the MT group was lower than that in the RAPA group (*p* < 0.05). No significant difference was found between the 3-MA and MT groups (*p* > 0.05) (Table 1). These results suggest that MT could improve autophagy-induced ovarian tissue injury, increase the number of primordial, primary, and sinus follicles, and reduce the number of atretic follicles.

### Effect of MT on serum sex hormone levels in rats

Serum MH and INH-B levels in each group were measured using ELISA. Serum AMH levels were highest in the control group and lowest in the model group; serum AMH levels in the RAPA group were higher than those in the model group (*p* < 0.05), and serum AMH and INH-B levels in the 3-MA and MT groups were higher than those in the RAPA group (*p* < 0.05). There was no significant difference in AMH and INH-B levels between the 3-MA and MT groups (*p* > 0.05). INH-B level in all groups showed a trend similar to that of AMH (Table 2).

**Table 2:** Serum AMH, INH-B, FSH, LH, E2, P, and T levels of rats in each group.

Group	n	AMH (ng/mL)	INH-B (pg/mL)	FSH (IU/L)	LH (mIU/mL)	E2 (pmoL/L)	P (ng/mL)	T (ng/mL)
Control group	19	2.76 ± 0.59	29.78 ± 3.15	7.06 ± 0.47	5.01 ± 0.47	128.69 ± 0.84	2.32 ± 0.39	0.22 ± 0.03
Model group	19	0.34 ± 0.07^a^	11.89 ± 2.93^a^	26.06 ± 3.74^a^	13.90 ± 2.68^a^	40.45 ± 1.87^a^	2.06 ± 0.59	0.25 ± 0.13
3-MA group	18	1.01 ± 0.10^a^^b^	21.50 ± 2.45^a^^b^	16.67 ± 1.43^a^^b^	7.88 ± 1.50^a^^b^	81.34 ± 2.70^a^^b^	2.25 ± 0.95	0.22 ± 0.06
RAPA group	19	0.47 ± 0.07^a^^b^^c^	14.00 ± 2.87^a^^b^^c^	22.10 ± 3.90^a^^b^^c^	10.50 ± 3.48^a^^b^^c^	62.83 ± 0.66^a^^b^^c^	2.03 ± 0.53	0.29 ± 0.06
MT group	18	1.09 ± 0.06^a^^b^^d^	21.12 ± 2.53^a^^b^^d^	16.06 ± 2.67^a^^b^^d^	7.07 ± 2.62^a^^b^^d^	79.98 ± 2.72^a^^b^^d^	2.21 ± 0.76	0.21 ± 0.07

a p compared with the control group, *p* < 0.05.

b p compared with the model group, *p* < 0.05.

c p compared with the 3-MA group, *p* < 0.05.

d p compared with RAPA group, *p* < 0.05.

Estrogen levels of FSH, LH, E2, P, and T were measured in rats. As a result, the levels of FSH and LH were found to be the lowest in the control group (*p* < 0.05) and highest in the model group (*p* < 0.05). Serum FSH and LH levels were lower in the RAPA group than the model group (*p* < 0.05), and serum FSH and LH levels were lower in the 3-MA and MT groups than the RAPA group (*p* < 0.05). No significant difference in FSH and LH levels was found between the 3-MA and MT groups (*p* > 0.05). E2 levels were the highest in the control group (*p* < 0.05) and lowest in the model group (*p* < 0.05). Serum E2 levels in the RAPA group were elevated compared with those in the model group (*p* < 0.05), and E2 levels in the MT and 3-MA groups were elevated compared with those in the RAPA group (*p* < 0.05). No significant difference in E2 levels was found between the 3-MA and MT groups (*p* > 0.05). Further, there was no significant difference in T and P levels among the groups (*p* > 0.05) (Table 2).

### Effect of MT on the PI3K/AKT/mTOR signaling pathway in rat ovarian tissues

**Fig. 2:**
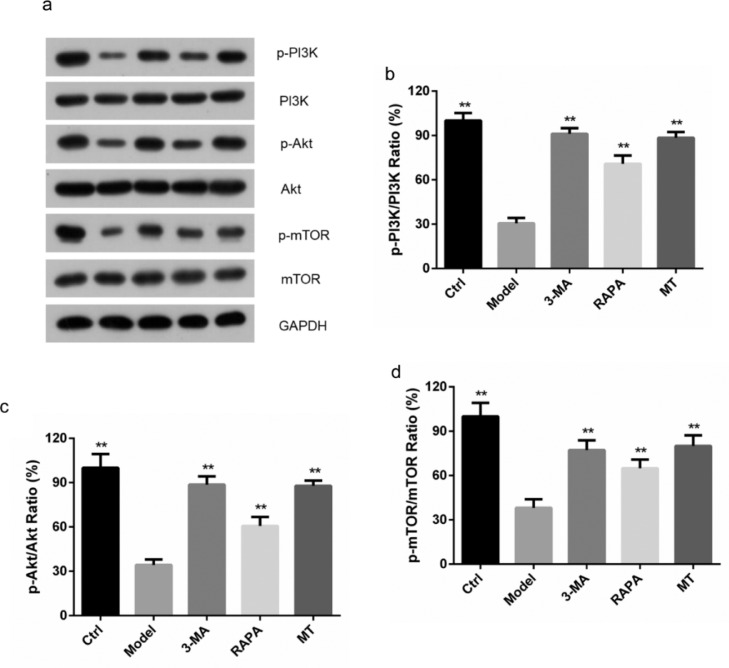
**Effect of MT on the PI3K/Akt/mTOR signaling pathway.** (a) Expression of the PI3K/AKT/mTOR pathway-related proteins, p-PI3K, p-Akt, p-mTOR proteins, and corresponding native proteins in the control, model, 3-MA, RAPA, and MT groups were detected by immunoblotting. (b‒d) Expression of the PI3K/AKT/mTOR pathway-related proteins, p-PI3K, p-Akt, p-mTOR proteins, and the corresponding native proteins were detected by ImageJ software (three experiments were independently performed).

The expression levels of the p-PI3K, p-AKT and p-mTOR protein were the highest in the control group (*p* < 0.05) and lowest in the model group (*p* < 0.05). p-PI3K, p-AKT, and p-mTOR protein expression levels were higher in the RAPA group than in the model group (*p* < 0.05). p-PI3K, p-AKT, and p-mTOR protein expression levels in the 3-MA and MT groups were higher than those in the model group (*p* < 0.05). AKT and p-mTOR protein expression levels were higher in the 3-MA and MT groups than in the RAPA group (*p* < 0.05). No statistical difference was found in protein expression levels between the 3-MA and MT groups (Fig. 2a–d). These results suggest that MT inhibits the PI3K/AKT/mTOR signaling pathway.

### MT has a protective effect on ovarian granulosa cells in rats with ovarian damage

**Fig. 3:**
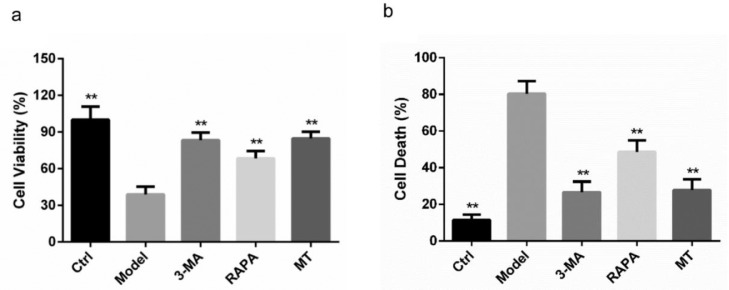
**The protective effect of MT on ovarian granulosa cells in each group of rats.** (a) The survival of rat ovarian granulosa cells in each group was detected by the CCK8 method. (b) The death of ovarian granulosa cells in each group was detected by the LDH method.

Based on the above experimental results, MT has a certain therapeutic effect on ovarian damaged rats and has an improvement and repair effect on their ovarian tissues. The authors of the present study isolated granulosa cells from rat ovarian tissues for a subsequent study. The isolated ovarian granulosa cells of each group were incubated together with various compounds, and ovarian granulosa cell viability was assayed using the CCK8 kit following incubation. Compared with the control group, the ovarian granulosa cell viability of rats in the model group was significantly decreased (*p* < 0.05), while the cell viability of the RAPA, 3-MA, and MT groups was significantly increased; there was no significant difference between the 3-MA and MT groups (Fig. 3a). The authors further detected cell mortality using the LDH method and found that the control group had the lowest cell mortality while the model group exhibited the highest cell mortality, which was significantly decreased in the RAPA, 3-MA, and MT groups; there was no significant difference between the 3-MA and MT groups (Fig. 3b).

### MT inhibits autophagic apoptosis of ovarian granulosa cells in rats with ovarian damage

**Fig. 4:**
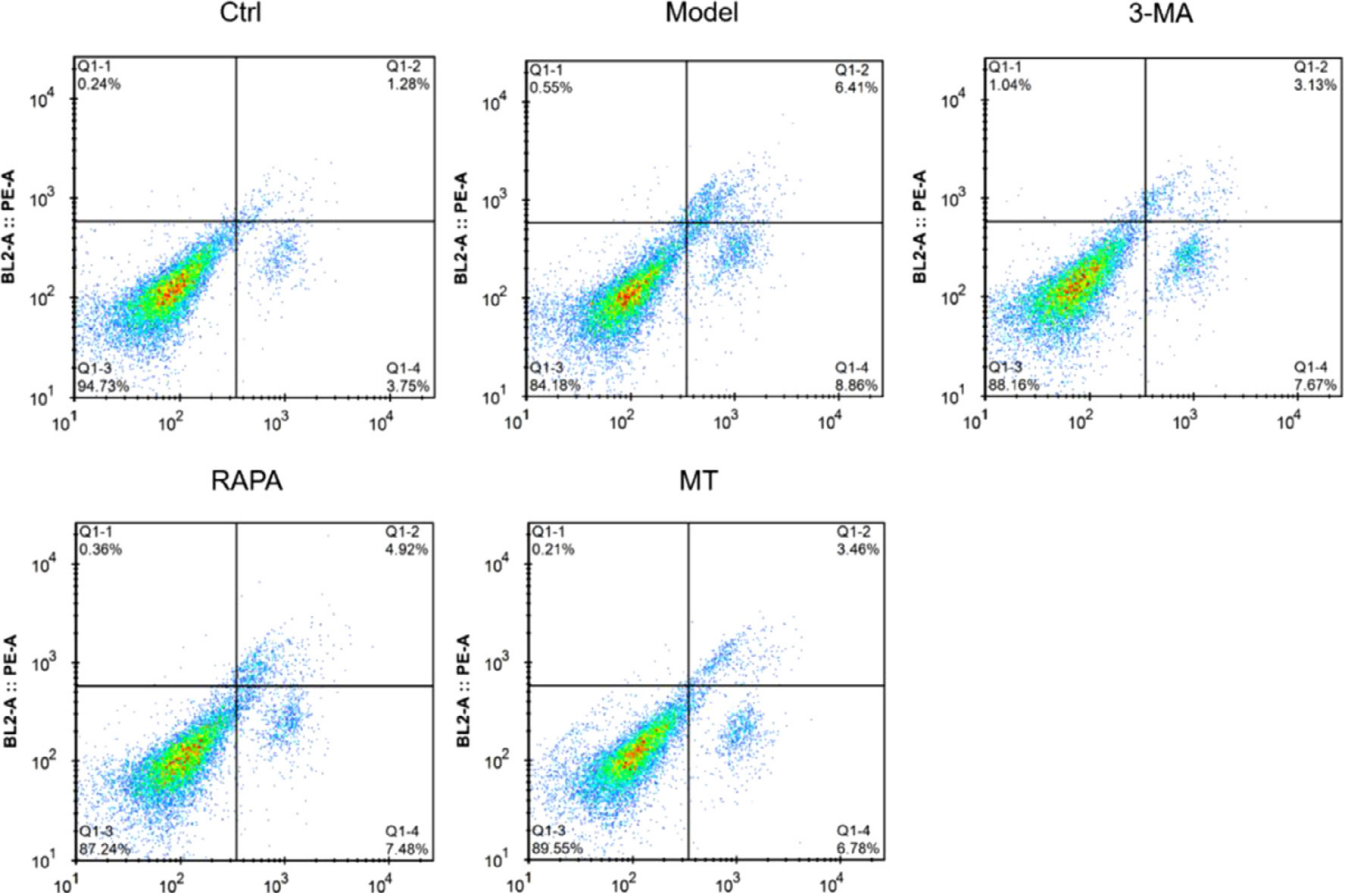
**Effect of MT on autophagic apoptosis of rat ovarian granulosa cells.** Flow cytometry was used to detect apoptosis in the ovarian granulosa cells of each group.

To determine whether the protective effect of MT on ovarian granulosa cells was achieved through the inhibition of autophagic apoptosis, the authors examined the apoptosis of well-isolated ovarian granulosa cells in each group by flow cytometry. The apoptosis rate was found to be significantly higher in all groups relative to the control group. The highest apoptosis rate of ovarian granulosa cells was found in the model group. Further, the apoptosis rate was significantly lower in the RAPA, 3-MA, and MT groups. No significant difference in apoptosis rate was found between the 3-MA and MT groups (Fig. 4). These results suggest that MT exerts its protective effect by inhibiting autophagic apoptosis of ovarian granulosa cells in ovarian damage rats.

### MT inhibits the expression of key proteins in the autophagic pathway

**Fig. 5:**
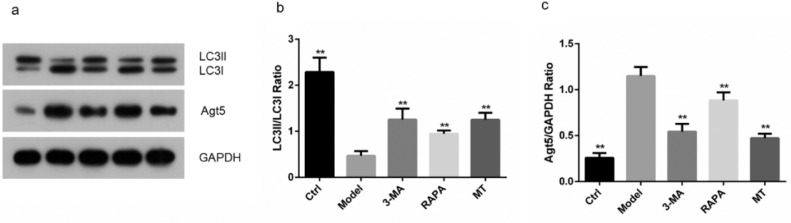
**Effect of MT on the expression of autophagy-related proteins.** (a) The expression of autophagy-associated proteins, LC3II/I and Agt5, was determined in ovarian granulosa cells by immunoblotting. (b‒c) The expression levels of autophagy-related proteins, LC3II/I and Agt5, were determined by ImageJ software (the experiments were independently performed three times).

The authors examined the expression of the autophagy-related proteins, LC3II, LC3I, and Agt5, in ovarian granulosa cells of rats in each group. The LC3II/I ratio was significantly higher and the expression level of the Agt5 protein was significantly upregulated in the ovarian granulosa cells of rats in the other groups compared with the control group (*p* < 0.05). Compared with the model group, the expression levels of the Agt5 protein and LC3II/I were significantly lower in the RAPA, 3-MA, and MT groups (*p* < 0.05). There was no significant difference in the levels of the Agt5 protein and LC3II/I between the 3-MA and MT groups (Fig. 5).

## Discussion

The ovary is a crucial reproductive organ in women as it is the site of egg production and excretion and is a key endocrine gland that secretes estrogen.^27^ As women age, ovarian function gradually declines, and the risk of POI increases significantly.^28^^,^^29^ The prevalence of POI in women under the age of 30 years is reported to be 0.1%, which increases to 1–2% in women aged 40 years.^3^ Furthermore, ovarian damage often leads to POI. Ovarian damage usually has a significant impact on individuals physically and mentally, causing major health challenges and psychological sequelae. Long-term sequelae include increased cardiovascular events, increased risk of dementia, early onset of osteoporosis, decreased cognitive function, reduced life expectancy, devastating psychological effects, infertility and sexual dysfunction, and increased mortality.^30^, ^31^, ^32^, ^33^, ^34^, ^35^, ^36^, ^37^ Therefore, it is important to identify appropriate strategies for the prevention and treatment of ovarian damage.

Patients with POI of different etiologies are currently treated clinically with hormone replacement therapy.^38^ MT, a hormone secreted by mammals and humans, is effective at reducing oxidative damage to cells, and its efficacy stems from its ability to scavenge free radicals directly and play a role as an antioxidant.^39^, ^40^, ^41^ MT has been reported to have a protective effect against POI induced by tretinoin polysaccharides.^42^ The present study's *in vivo* experiments based on ovarian damage rats also revealed a protective effect of MT in ovarian damage rats, which was achieved by inhibiting the PI3K/AKT/mTOR autophagy pathway. In the current study, ovarian damage rats exhibited reduced follicle-stimulating hormone and luteinizing hormone levels and increased E2, ovarian volume, and endometrial thickness after treatment. Such findings support the protective effect of MT against ovarian damage. Follicle-stimulating hormone levels above 10 IU/L are known to be associated with reduced ovarian reserve. In the current study, although follicle-stimulating hormone levels did not decrease to a level below 10 IU/L, MT caused a significant change in follicle-stimulating hormone levels.

RAPA is an inhibitor of mTOR and controls apoptosis and proliferation by regulating the levels of biological signals, such as amino acids, glycans, and insulin.^43^ Adhikari et al. reported that the knockdown of tuberous sclerosis complexes 1 and 2 in mouse oocytes resulted in elevated mTOR1 activity and rapid conversion of primordial follicles to growing follicles, leading to premature ovarian follicular failure and POI.^44^ This study also suggests that RAPA may play a crucial role in regulating the recruitment, differentiation, and proliferation of primordial follicles. 3-MA is an inhibitor of the autophagic pathway and acts by blocking the formation of autophagosomes and inhibiting PI3K kinase during the chelation step.^45^ In this study, the authors measured the levels of estrogens, such as ovarian wet weight, ovarian index, AMH, INH-B, and FSH in rats. The numbers of primordial follicles, primary follicles, sinus follicles, and atretic follicles were also recorded in each group of rats. Based on the above results, the authors found that MT showed similar effects to 3-MA in the reduction of ovarian wet weight and ovarian index. AMH, INH-B, and E2 levels were significantly higher in both the 3-MA and MT groups than in the RAPA group. The levels of LH and FSH in the 3-MA and MT groups were lower than those in the RAPA group. There was no significant difference in AMH, INH-B, LH, T, E2, and P levels between the 3-MA and MT groups (*p* > 0.05). MT was also found to have a similar effect on the number of primordial follicles, primary follicles, sinus follicles, and atretic follicles. Such a finding explains the protective effect of MT against ovarian damage. Western blot assay also confirmed that MT could increase the expression of p-PI3K, p-AKT, and p-mTOR in ovarian tissues of rats with ovarian damage, with similar effects as 3-MA. Moreover, *in vitro* cellular assays showed that MT protected the ovaries of ovarian damage rats by inhibiting the apoptosis of ovarian granulosa cells.

This study also has some limitations. The authors have conducted in-depth research on the ovarian protective effect of MT through animal experiments and preliminarily explored its protective mechanism. However, it is not clear which gene target MT acts on to protect the damaged ovary and the relationship between this target and the autophagy pathway, which needs to be further explored.

## Conclusion

In conclusion, the authors confirmed the protective effect of MT against ovarian damage *in vivo* and *in vitro*. The role of 3-MA in ovarian damage is similar to that of MT and exerts its protective effect against ovarian damage by inhibiting autophagic apoptosis through the regulation of the PI3K/AKT/mTOR signaling pathway.

## Funding

This research did not receive any specific grant from funding agencies in the public, commercial, or not-for-profit sectors.

## Conflicts of interest

The authors declare no conflicts of interest.

## CRediT authorship contribution statement

**Yan Liu:** Conceptualization, Investigation, Writing – original draft. **Xiaohe Zhu:** Data curation. **Chunli Wu:** Data curation. **Yan Lang:** Formal analysis, Validation. **Wenjie Zhao:** Formal analysis, Validation. **Yanmin Li:** Conceptualization, Investigation, Writing – review & editing.

## References

[1] Jaillard S., Bell K., Akloul L., Walton K., McElreavy K., Stocker W.A. (2020). New insights into the genetic basis of premature ovarian insufficiency: novel causative variants and candidate genes revealed by genomic sequencing. Maturitas.

[2] Sharif K., Watad A., Bridgewood C., Kanduc D., Amital H., Shoenfeld Y. (2019). Insights into the autoimmune aspect of premature ovarian insufficiency. Best Pract Res Clin Endocrinol Metab.

[3] Rudnicka E., Kruszewska J., Klicka K., Kowalczyk J., Grymowicz M., Skórska J. (2018). Premature ovarian insufficiency - aetiopathology, epidemiology, and diagnostic evaluation. Prz Menopauzalny.

[4] Huang Y., Hu C., Ye H., Luo R., Fu X., Li X. (2019). Inflamm-aging: a new mechanism affecting premature ovarian insufficiency. J Immunol Res.

[5] Fraison E., Crawford G., Casper G., Harris V., Ledger W. (2019). Pregnancy following diagnosis of premature ovarian insufficiency: a systematic review. Reprod Biomed Online.

[6] Jiao X., Zhang H., Ke H., Zhang J., Cheng L., Liu Y. (2017). Premature ovarian insufficiency: phenotypic characterization within different etiologies. J Clin Endocrinol Metab.

[7] Nguyen H.H., Milat F., Vincent A. (2017). Premature ovarian insufficiency in general practice: Meeting the needs of women. Aust Fam Phys.

[8] Ishizuka B. (2021). Current understanding of the etiology, symptomatology, and treatment options in premature ovarian insufficiency (POI). Front Endocrinol.

[9] Fruzzetti F., Palla G., Gambacciani M., Simoncini T. (2020). Tailored hormonal approach in women with premature ovarian insufficiency. Climacteric.

[10] Kokcu A. (2010). Premature ovarian failure from current perspective. Gynecol Endocrinol.

[11] Taylor A.P., Lee H., Webb M.L., Joffe H., Finkelstein JS. (2016). Effects of testosterone and estradiol deficiency on vasomotor symptoms in hypogonadal men. J Clin Endocrinol Metab.

[12] Vargas K.G., Milic J., Zaciragic A., Wen K.X, Jaspers L., Nano J. (2016). The functions of estrogen receptor beta in the female brain: a systematic review. Maturitas.

[13] Gallagher J.S., Missmer S.A., Hornstein M.D., Laufer M.R., Gordon C.M., DiVasta A.D. (2018). Long-term effects of gonadotropin-releasing hormone agonists and add-back in adolescent endometriosis. J Pediatr Adolesc Gynecol.

[14] Cannarella R., Barbagallo F., Condorelli R.A., Aversa A., La Vignera S., Calogero A.E. (2019). Osteoporosis from an endocrine perspective: the role of hormonal changes in the elderly. J Clin Med.

[15] Kander M.C., Cui Y., Liu Z. (2017). Gender difference in oxidative stress: a new look at the mechanisms for cardiovascular diseases. J Cell Mol Med.

[16] Sullivan S.D., Sarrel P.M., Nelson LM. (2016). Hormone replacement therapy in young women with primary ovarian insufficiency and early menopause. Fertil Steril.

[17] Tamura H., Jozaki M., Tanabe M., Shirafuta Y., Mihara Y., Shinagawa M. (2020). Importance of melatonin in assisted reproductive technology and ovarian aging. Int J Mol Sci.

[18] Sagrillo-Fagundes L., Assunção Salustiano E.M., Yen P.W., Soliman A., Vaillancourt C. (2016). Melatonin in pregnancy: effects on brain development and CNS programming disorders. Curr Pharm Des.

[19] Minguini I.P., Luquetti C.M., Baracat M.C.P., Maganhin C.C., Nunes C.O., Simões R.S. (2019). Melatonin effects on ovarian follicular cells: a systematic review. Rev Assoc Med Bras.

[20] Shiroma M.E., Botelho N.M., Damous L.L., Baracat E.C., Soares J.M. (2016). Melatonin influence in ovary transplantation: systematic review. J Ovarian Res.

[21] Ferreira C.S., Carvalho K.C., Maganhin C.C., Paiotti A.P.R., Oshima C.T.F., Simões M.J. (2016). Does melatonin influence the apoptosis in rat uterus of animals exposed to continuous light?. Apoptosis.

[22] Ferreira C.S., Maganhin C.C., Simões R.S., Girão M., Baracat E.C., Soares J.M. (2010). Melatonin: cell death modulator. Rev Assoc Med Bras.

[23] Zhang L., Liang YJ. (2014). Melatonin regulates ovarian function: an update. Zhonghua Nan Ke Xue.

[24] Cipolla-Neto J., Amaral F.G., Soares J.M., Gallo C.C., Furtado A., Cavaco J.E. (2022). The crosstalk between melatonin and sex steroid hormones. Neuroendocrinology.

[25] Soares J.M., Masana M.I., Erşahin C., Dubocovich ML. (2003). Functional melatonin receptors in rat ovaries at various stages of the estrous cycle. J Pharmacol Exp Ther.

[26] Maganhin C.C., Baracat M.C.P., Carvalho K.C., Seganfredo I.B., Luquetti C.M., Simões R.S. (2020). Evidence that melatonin increases inhibin beta-A and follistatin gene expression in ovaries of pinealectomized rats. Reprod Sci.

[27] Barakat R., Oakley O., Kim H., Jin J., Ko C.J. (2016). Extra-gonadal sites of estrogen biosynthesis and function. BMB Rep.

[28] Bachelot A., Nicolas C., Bidet M., Dulon J., Leban M., Golmard J.L. (2017). Long-term outcome of ovarian function in women with intermittent premature ovarian insufficiency. Clin Endocrinol.

[29] Podfigurna-Stopa A., Czyzyk A., Grymowicz M., Smolarczyk R., Katulski K., Czajkowski K. (2016). Premature ovarian insufficiency: the context of long-term effects. J Endocrinol Invest.

[30] Tsiligiannis S., Panay N., Stevenson JC. (2019). Premature Ovarian insufficiency and long-term health consequences. Curr Vasc Pharmacol.

[31] Panay N., Anderson R.A., Nappi R.E., Vincent A.J., Vujovic S., Webber L. (2020). Premature ovarian insufficiency: an international menopause society white paper. Climacteric.

[32] Roeters van Lennep J.E., Heida K.Y., Bots M.L., Hoek A. (2016). Cardiovascular disease risk in women with premature ovarian insufficiency: a systematic review and meta-analysis. Eur J Prev Cardiol.

[33] Li N., Liu L. (2018). Mechanism of resveratrol in improving ovarian function in a rat model of premature ovarian insufficiency. J Obstet Gynaecol Res.

[34] Shestakova I.G., Radzinsky V.E., Khamoshina MB. (2016). Occult form of premature ovarian insufficiency. Gynecol Endocrinol.

[35] Nappi R.E., Cucinella L., Martini E., Rossi M., Tiranini L., Martella S. (2019). Sexuality in premature ovarian insufficiency. Climacteric.

[36] Podfigurna A., Stellmach A., Szeliga A., Czyzyk A., Meczekalski B. (2018). Metabolic profile of patients with premature ovarian insufficiency. J Clin Med.

[37] Szeliga A., Maciejewska-Jeske M., Męczekalski B. (2018). Bone health and evaluation of bone mineral density in patients with premature ovarian insufficiency. Prz Menopauzalny.

[38] Machura P., Grymowicz M., Rudnicka E., Pięta W., Calik-Ksepka A., Skórska J. (2018). Premature ovarian insufficiency - hormone replacement therapy and management of long-term consequences. Prz Menopauzalny.

[39] Deng S.L., Sun T.C., Yu K., Wang Z.P., Zhang B.L., Zhang Y. (2017). Melatonin reduces oxidative damage and upregulates heat shock protein 90 expression in cryopreserved human semen. Free Radic Biol Med.

[40] Reiter R.J., Tan D.X., Rosales-Corral S., Galano A., Zhou X.J., Xu B. (2018). Mitochondria: central organelles for melatonin's antioxidant and anti-aging actions. Molecules.

[41] Reiter R.J., Rosales-Corral S., Tan D.X., Jou M.J., Galano A., Xu B. (2017). Melatonin as a mitochondria-targeted antioxidant: one of evolution's best ideas. Cell Mol Life Sci.

[42] Emerick B., Schleiniger G., Boman BM. (2017). A kinetic model to study the regulation of beta-catenin, APC, and Axin in the human colonic crypt. J Math Biol.

[43] Dobashi Y., Watanabe Y., Miwa C., Suzuki S., Koyama S. (2011). Mammalian target of rapamycin: a central node of complex signaling cascades. Int J Clin Exp Pathol.

[44] Tong Y., Li F., Lu Y., Cao Y., Gao J., Liu J. (2013). Rapamycin-sensitive mTORC1 signaling is involved in physiological primordial follicle activation in mouse ovary. Mol Reprod Dev.

[45] Guo S., Yang B., Liu H., Shao J., Zhang Q. (2019). Experimental study of autophagy inhibitor 3-MA combined with PI3K/mTOR dual inhibitor NVP-BEZ235 promote apoptosis of cervical cancer cells. J Youjiang Med Univ Natl.

